# Effect of Axial Force on the Performance of Micromachined Vibratory Rate Gyroscopes

**DOI:** 10.3390/s110100296

**Published:** 2010-12-29

**Authors:** Zhanqiang Hou, Dingbang Xiao, Xuezhong Wu, Peitao Dong, Zhihua Chen, Zhengyi Niu, Xu Zhang

**Affiliations:** College of Mechanical Engineering and Automation, National University of Defense Technology, Changsha, Hunan Province, 410073, China; E-Mails: houzhanqiang1022@163.com (Z.H.) dingbangxiao@yahoo.com.cn (D.X.); ptdong@nudt.edu.cn (P.D.); zhihuachen@nudt.edu.cn (Z.C.); zyniu163@163.com (Z.N.); xuzhang@163.com (X.Z.)

**Keywords:** micromachined vibratory gyroscope, thermal stress, resonant frequency, slanted beam, temperature characteristics, anodic bonding

## Abstract

It is reported in the published literature that the resonant frequency of a silicon micromachined gyroscope decreases linearly with increasing temperature. However, when the axial force is considerable, the resonant frequency might increase as the temperature increases. The axial force is mainly induced by thermal stress due to the mismatch between the thermal expansion coefficients of the structure and substrate. In this paper, two types of micromachined suspended vibratory gyroscopes with slanted beams were proposed to evaluate the effect of the axial force. One type was suspended with a clamped-free (C-F) beam and the other one was suspended with a clamped-clamped (C-C) beam. Their drive modes are the bending of the slanted beam, and their sense modes are the torsion of the slanted beam. The relationships between the resonant frequencies of the two types were developed. The prototypes were packaged by vacuum under 0.1 mbar and an analytical solution for the axial force effect on the resonant frequency was obtained. The temperature dependent performances of the operated mode responses of the micromachined gyroscopes were measured. The experimental values of the temperature coefficients of resonant frequencies (TCF) due to axial force were 101.5 ppm/°C for the drive mode and 21.6 ppm/°C for the sense mode. The axial force has a great influence on the modal frequency of the micromachined gyroscopes suspended with a C-C beam, especially for the flexure mode. The quality factors of the operated modes decreased with increasing temperature, and changed drastically when the micromachined gyroscopes worked at higher temperatures.

## 1. Introduction

Micromachined vibratory gyroscopes are of great interest due to their small size, low cost, batch fabrication and high reliability and their performance has been constantly improving over the past decades. They are becoming a viable alternative to expensive and bulky conventional gyroscopes and are widely used in many applications ranging from automobile stability to inertial navigation, such as roll-over detection and prevention, image stabilization in digital cameras and camcorders, computer gaming industry, micro-satellites, micro-robotics, guided guns and missiles [1–6]. There is however still a lack of high performance, low cost products on the market to meet the on-going demand. The challenge has been in identifying what and how the factors affect the performance of micromachined gyroscopes. It is proven that the performance limit of micromachined gyroscopes is on the order of 0.01–0.001°/h [7–9], but in practice such levels of performance have never been achieved in any available gyroscopes.

The basic architecture of a micromachined vibratory gyroscope is comprised of a drive mode and a sense mode. The drive mode generates and maintains a constant linear or angular momentum, and the sense mode measures the Coriolis force induced by drive vibration and angular rate input. Therefore, the characteristics of the drive and sense mode are the most crucial dynamical parameters for designing a good performance gyroscope. Phase Lock Loop and Automatic Gain Control are common and convenient methods to achieve a stable drive mode amplitude, so the resonant frequency is becoming the definitely parameter determining the performance of the gyroscope.

Silicon and Pyrex glass in bulk micromachining and SOI in surface micromachining are the most commonly used materials in MEMS vibratory gyroscopes [10–12]. Temperature effects on micromachined vibratory gyroscopes are variable and severe, because silicon and silicon dioxide are sensitive to environmental temperature fluctuations. Extensive research has emphatically focused on the mechanical thermal noise, mechanical loss, residual stress and thermal stress caused by temperature variations [13–16].

Common suspension structures utilized in micromachined vibratory gyroscopes are designed with clamped-clamped suspension beams including crab-leg suspensions, H-type suspensions, and U-beam suspensions [17]. When the temperature changes, there will be a thermal stress due to the mismatch of the thermal expansion coefficients between the silicon structure and the substrate. This can cause an axial force into the clamped-clamped suspension beams, which will lead to a resonance frequency shift. The frequency shift due to thermal stress is simulated by finite element analysis in [16], but an exact analytical model has not been mentioned. It was proven in published literature that the resonant frequency decreased linearly as the temperature increased [18–21]. However, in our research, the resonant frequency of the drive mode increased as the temperature increased. It has been found that a positive change in frequency was observed due to a considerable axial force.

In this paper, two types of silicon gyroscopes with slanted suspension beams are introduced. Our work focuses on the effect of axial force on the performance of the micromachined gyroscopes. The relationship of the resonant frequency between the two types was developed. An analytical solution for the axial force effect on the resonant frequency was obtained. The temperature dependent performance of the operated mode response was studied experimentally.

## 2. Structures of the Micromachined Gyroscopes

**Figure 1.**
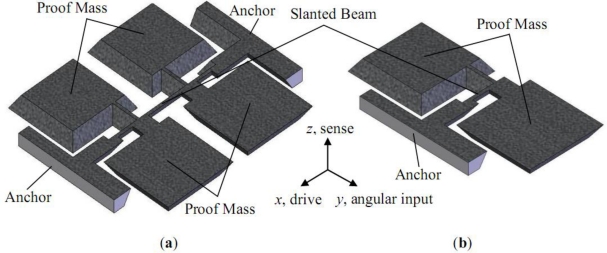
Schematic sketch of the gyroscopes. **(a)** clamped-clamped beam **(b)** clamped-free beam.

The schematic sketches of the two presented types of the micromachined gyroscopes are shown in Figure 1. The first type is suspended with a clamped-clamped beam, which was first reported in [22]. The second type is half of the first one. The proof mass was suspended with a slanted cantilever. Its structure is similar to that reported in [23].

**Figure 2.**
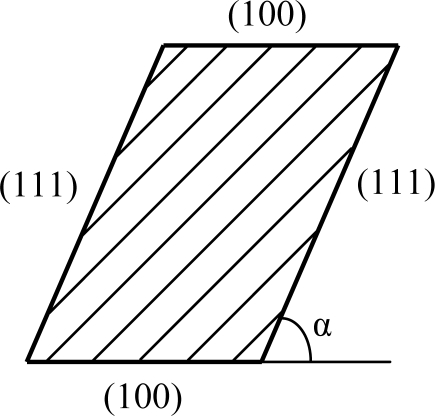
Cross section of the slanted beam.

The cross section of the slanted suspension beams is shown in Figure 2. It is formed by a (100) and (111) crystal plane and the angle between the two planes, α, is 54.74°.

The prototypes of the two types of gyroscopes were fabricated on one n-type (100) silicon wafer to minimize the differences between them. The silicon structure was fabricated by anisotropic etching and then anodic bonding to the Pyrex glass deposited with electrodes. Vacuum packaging under 0.1 mbar was implemented at the die level.

## 3. Working Principle of the Micromachined Gyroscopes

The micromachined vibratory gyroscopes employ a combination of proof mass and flexures to form a resonator in both the drive and sense directions. Each of the resonators is modeled as a spring-mass-damper system and described as a second-order differential equation. Considering the proof mass of the drive and sense resonator is common used, the motion equation of the micromachined gyroscope can be described as:

(1) $$\begin{array}{l} m\ddot{x}+c_{d}\;\dot{x}+k_{d}\;x=F_{d}\\ m\ddot{y}+c_{s}\;\dot{y}+k_{s}\;y=-2m\Omega \dot{x} \end{array}$$

where *m* denotes the mass of the proof mass; *c_d_*, *c_s_* denote the damping factor of the drive and sense resonator; *k_d_*, *k_s_* denote the stiffness of the drive and sense resonator; *x*, *y* denote the displacement in the drive and sense direction; Ω is the angular rate input.

### 3.1. Drive Mode

**Figure 3.**
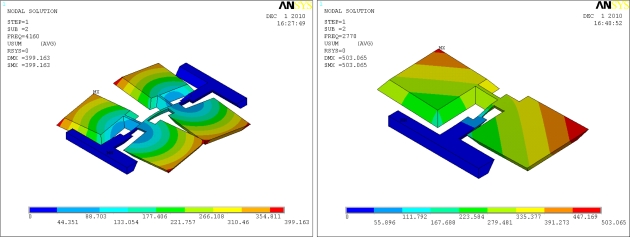
The drive modes of the micromachined gyroscopes.

Since the Coriolis effect is based on conservation of momentum, every gyroscope requires a mechanical oscillator to generate the momentum, which is called the drive mode oscillator. The drive mode oscillator commonly operates on self resonance to minimize the excitation voltages, and achieves a stable amplitude and phase by the use of Phase Lock Loop and Automatic Gain Control methods. The drive modes of the introduced gyroscopes are the flexural vibrations of the slanted beams, as shown in Figure 3.

### 3.2. Sense Mode

**Figure 4.**
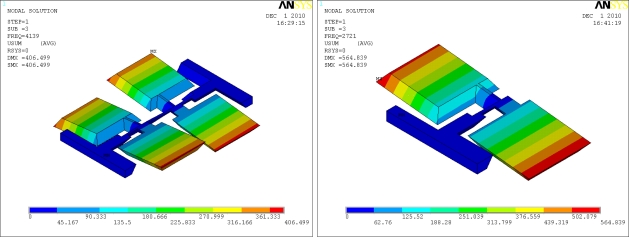
The sense modes of the micromachined gyroscopes.

The sense mode is designed to detect the Coriolis effect. With an angular rate input, there will be a sinusoidal oscillation in the sense direction due to the Coriolis force. The sense modes of the introduced gyroscopes are the torsional vibrations of the slanted beams, as shown in Figure 4.

## 4. Resonant Frequencies of the Micromachined Gyroscopes

**Figure 5.**
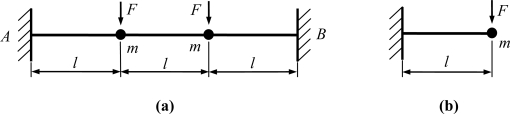
Kinematic analysis of the drive mode: flexural vibration of the slanted beam. **(a)** clamped-clamped **(b)** clamped-free.

The drive modes of the introduced gyroscopes are the flexural vibrations of the slanted beams, which are normally excited by supplying a sinusoidal signal plus DC bias voltage. The equivalent kinematic models are shown in Figure 5.

In addition, the beams follow the Euler-Bernoulli beam theory. Considering two types of gyroscopes driven by same conditions, the stiffness of the clamped-clamped beam *k*_*d*C-C_ and the stiffness of the clamped-free beam *k*_*d*C-F_ can be described as:

(2) $$k_{d\text{C-C}}=\frac{6\mathrm{EI}}{l^{3}}$$

(3) $$k_{d\text{C-F}}=\frac{3\mathrm{EI}}{l^{3}}$$

where *E* is the Young’s modulus of silicon, *I* is the moment of inertia, *l* is the length of the suspended beam.

Because the gyroscopes are vacuum packaged, the resonant frequency *ω_n_* can be considered as the natural frequency. Thus, the resonant frequencies of drive modes can be given by:

(4) $$\omega_{d\text{C-C}}=\sqrt{2}\omega_{d\text{C-F}}=\sqrt{\frac{k}{m}}=\sqrt{\frac{6\mathrm{EI}}{{\mathrm{ml}}^{3}}}$$

**Figure 6.**
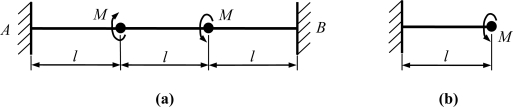
Kinematic analysis of the sense mode: torsional vibration of the slanted beam. (**a**) clamped-clamped (**b**) clamped-free.

From the Equation (4), we can conclude that the resonant frequency of the drive mode of the C-C beam is $\sqrt{2}$ times as high as that of the C-F beam. The sense modes of the introduced gyroscopes are the torsional vibrations of the slanted beams. The equivalent kinematic models are shown in Figure 6.

Similar to the drive mode oscillator, the stiffness of the clamped-clamped beam *k*_*s*C-C_ and the stiffness of the clamped-free beam *k_s_*_C-F_ can be described as:

(5) $$k_{s\text{C-C}}=\frac{3{\mathrm{GI}}_{p}}{l}$$

(6) $$k_{s\text{C-F}}=\frac{{\mathrm{GI}}_{p}}{l}$$

where *G* is the shear modulus of silicon, *I_p_* is the area moment of inertia.

The resonant frequencies of sense modes for two types of gyroscopes can be given by:

(7) $$\omega_{\text{sC-C}}=\sqrt{3}\omega_{\text{sC-F}}=\sqrt{\frac{3{\mathrm{GI}}_{p}}{\mathrm{Jl}}}$$

where *J* is the moment of inertia of the proof mass with respect to the rotation axis.

## 5. Effect of Axial Force on the Resonant Frequency of the Micromachined Gyroscopes

Silicon is sensitive to temperature fluctuations. The temperature effect of the Young’s modulus was analyzed and experimentally verified in previous researches. It has been proven that there was a strong linear dependence of the Young’s modulus with temperature. The Young’s modulus can be expressed as [24]:

(8) $$E\left(T_{1}\right)=E\left(T_{0}\right)+K_{E}\cdot E\left(T_{0}\right)\cdot \left(T_{1}-T_{0}\right)$$

where *K_E_* ≈ −5 × 10^−5^ N/MT denotes the temperature coefficient of the Young’s modulus of silicon; *E*(*T_0_*), *E*(*T_1_*) is the Young’s modulus at the temperature *T_0_*, *T_1_* respectively.

The substrate of the micromachined gyroscope is Pyrex glass, its thermal expansion coefficient is 3.3 × 10^−6^ K^−1^ [25]. The suspension beam is made of single crystal silicon, its thermal expansion coefficient is 2.6 × 10^−6^ K^−1^ [26]. The difference of the thermal expansion coefficients between structure and substrate will bring an axial force to the clamped-clamped suspended beam.

**Figure 7.**
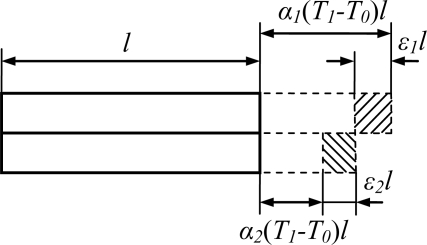
Thermal stress induced between two bonded materials when temperature was changed from *T_0_* to *T_1_*.

The two ends of the slanted beam are anodically bonded to the substrate. It can be considered as two rods fastened to each other with identical lengths *l* but different thermal expansion coefficients *α_1_, α_2_* (shown in Figure 7). When the temperature changes from *T_0_* to *T_1_*, the thermal stress in the suspension beam is given by Equation (9), assuming no relative displacement and bending between them [27]:

(9) $$\sigma =\frac{\alpha_{1}E\left(T_{1}\right)\cdot \left(T_{1}-T_{0}\right)\left(1-\frac{\alpha_{1}}{\alpha_{2}}\right)}{1+\frac{A_{1}\cdot E\left(T_{1}\right)}{A_{2}\cdot E_{1}\left(T_{1}\right)}}$$

where *E*, *E_1_* are the Young’s modulus of silicon and pyrex glass; *A_1_*, *A_2_* are the area of the suspended beam and substrate. *A_1_*, *A_2_*, so the Equation (9) can be simplified as:

(10) $$\sigma =\alpha_{1}E\left(T_{1}\right)\cdot \left(T_{1}-T_{0}\right)\left(1-\frac{\alpha_{1}}{\alpha_{2}}\right)$$

The natural frequency is affected by moment of inertia, shear deformation and axial force. As the size of the beam in length is much larger than that in width and height, the torsional inertia, shear deformation is negligible. The relationship between the natural frequency of the drive mode *ω*_*d*C-C_ and the axial force *N* can be described by [28]:

(11) $$\omega_{d\text{C-C}}=\omega_{0}\sqrt{1+\frac{9{\mathrm{Nl}}^{2}}{\pi^{2}\mathrm{EI}}}$$

Substituting (10) into (11), and considering *N* = *σA*, the natural frequency is:

(12) $$\omega_{d\text{C-C}}\left(T\right)=\sqrt{1+\frac{9\alpha_{1}\left(T-T_{0}\right)\left(1-\alpha_{1}/\alpha_{2}\right)\cdot A_{1}l^{2}}{\pi^{2}I}}\cdot \omega_{d\text{C-C}}\left(T_{0}\right)$$

The suspension beam was modeled using Euler-Bernoulli theory. When the axial force exerts on the beam, the distance among the cross sections and the interactive forces among the molecules will be changed. As a result, the shear modulus will be changed. The relationship between the shear modulus and the axial force may be denoted as a scale factor, similarly to the friction coefficient. So the shear modulus can be expressed as:

(13) $$G\left(T\right)=\left(1+\alpha_{N}\cdot N\right)\cdot G\left(T_{0}\right)$$

where, *α_N_* is the scale factor of the shear modulus affected by the axial force. The natural frequency of the sense mode can be described by:

(14) $$\omega_{s\text{C-C}}\;\left(T\right)=\sqrt{1+\alpha_{N}\cdot \alpha_{1}E\left(T\right)\left(T-T_{0}\right)\left(1-\alpha_{1}/\alpha_{2}\right)\cdot A_{1}}\cdot \omega_{s\text{C-C}}\;\left(T_{0}\right)$$

## 6. Experimental Setup and Results

**Figure 8.**
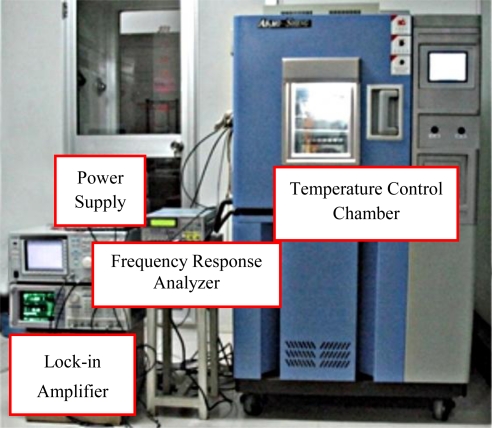
The temperature experimental setup of the gyroscopes.

In order to validate the effect of axial force, the two types of the micromachined gyroscopes were fabricated on one silicon wafer. The results in a good coherence between them. The temperature tests were performed using a temperature control chamber. The power supply, control signal and output signal were connected to the chamber (shown in Figure 8).

**Figure 9.**
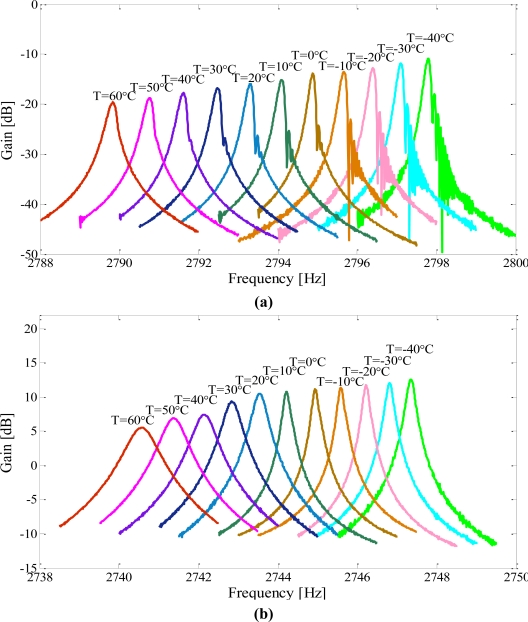
The frequency response characteristics of the gyroscope suspended with a C-F beam at different temperature levels, when operating at the same input conditions. **(a)** drive mode **(b)** sense mode.

**Figure 10.**
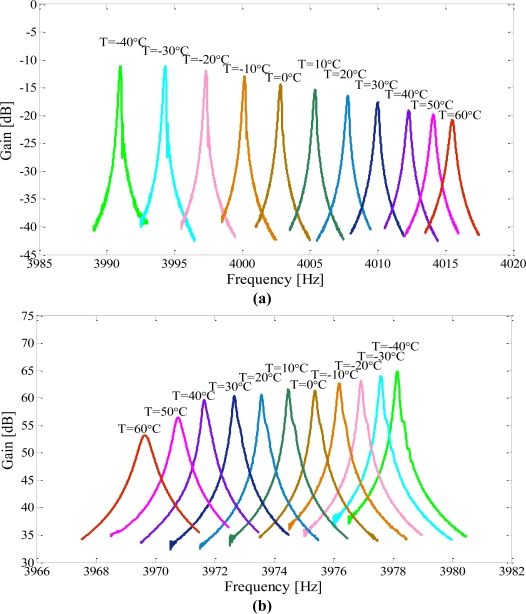
The frequency response characteristics of the gyroscope suspended with a C-C beam at different temperature levels, when operating at the same input conditions. (**a**) drive mode (**b**) sense mode.

The temperature was raised up from −40 °C to 60 °C at 10 °C intervals and each temperature point was maintained for 20 min. The frequency responses of the drive modes and sense modes were tested and recorded by a frequency response analyzer (NF FRA5087). Then the data was imported and plotted in Matlab. The outputs for the drive and sense modes of the micromachined gyroscope suspended with a C-F beam are shown in Figure 9(a,b); the outputs for that of the micromachined gyroscope suspended with a C-C beam are shown in Figure 10(a,b) respectively.

As shown in Figures 9(a,b), the resonant frequencies and the magnitudes of both the drive mode and the sense mode were decreased with increasing temperature in the C-F suspension beam type. This drift can mainly be attributed to the increased gain of the drive and sense oscillations as a result of softening effect on Young’s modulus.

The temperature dependent performances of the second type gyroscope were tested. The results are shown in Figures 10(a,b). The resonant frequency of the sense mode decreased with increasing temperature, while the resonant frequency of the drive mode was increased with increasing temperature. The change trends were just opposite to each other as external temperature variations. Also the gain of the output has the same change trend with the resonant frequency.

**Figure 11.**
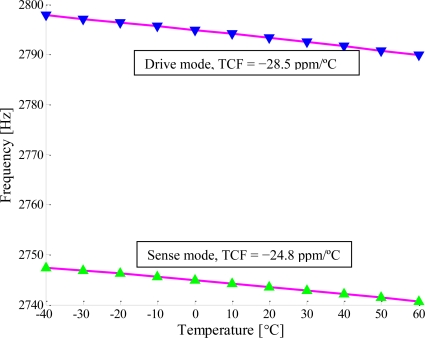
Temperature coefficients of the resonant frequency of the first type gyroscope suspended with a C-F beam.

**Figure 12.**
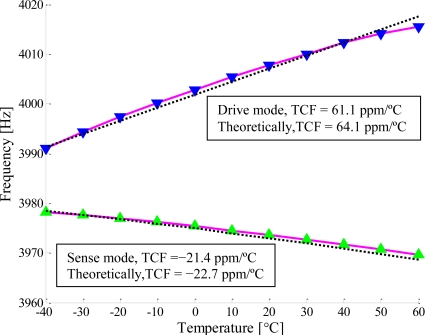
Temperature coefficients of the resonant frequency of the second type gyroscope suspended with a C-C beam.

The relationships between the resonant frequencies and the temperature for the two type gyroscopes are shown in Figure 11 and Figure 12. For the first type, the temperature coefficients of the resonant frequency (TCF) were measured to be −28.5 ppm/°C for the drive mode and −24.8 ppm/°C for the sense mode (shown in Figure 11). However, for the second type, the TCF were measured to be 61.1 ppm/°C for the drive mode and −21.4 ppm/°C for the sense mode (shown in Figure 12). From the Equations (4) and (7), we can get the temperature coefficients of the resonant frequencies for the drive mode and sense mode are −40.4 ppm/°C and −43.0 ppm/°C if the axial force is not considered. The effects of the axial force on the resonant frequencies for the drive and sense oscillations were given by Equations (12) and (14) respectively. The TCF are linearly fitted to be 64.1 ppm/°C for the drive mode and −22.7 ppm/°C for the sense mode, as is shown with dashed lines in Figure 12, where the scale factor, *α_N_*, obtained by the experimental data is −2.13e−2. The TCF due to axial force were 101.5 ppm/°C and 21.6 ppm/°C for the drive mode and the sense mode respectively.

**Figure 13.**
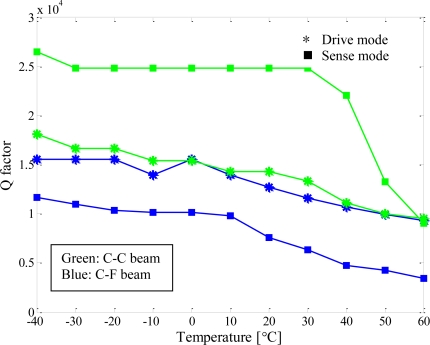
Experimental study of the quality factors of the presented micromachined gyroscopes.

In addition, the quality factors of the presented gyroscopes were characterized at different temperatures, as shown in Figure 13. The quality factors of the operated modes decreased with increasing temperature, and changed drastically when the gyroscopes worked at higher temperatures. The probable cause was that the die attachment was softened as the temperature increased.

## 7. Conclusions

In this work, two types of micromachined vibratory gyroscopes suspended with slanted beams are presented. One type is suspended with a clamped-free beam and the other one is suspended with a clamped-clamped beam. The temperature characterization of their operated mode response was tested and analyzed. For the C-F beam type, the TCF were measured to be −28.5 ppm/°C for the drive mode and −24.8 ppm/°C for the sense mode. Thus, the theoretical TCF of the C-C beam type were derived to be −40.4 ppm/°C and −43.0 ppm/°C for the drive and sense mode respectively. However, the TCF were measured to be 61.1 ppm/°C for the drive mode and −21.4 ppm/°C for the sense mode, confirming the analytical values of 64.1 ppm/°C and −22.7 ppm/°C. The differences between the analytical values and the experimental results are mainly caused by shear deformation, moment of inertia and nonlinear effects. Comparing with the theoretical values of −40.4 ppm/°C and −43.0 ppm/°C, the TCF due to axial force were 101.5 ppm/°C and 21.6 ppm/°C for the drive mode and sense mode respectively. The axial force has a great influence on the performance of the micromachined gyroscopes suspended with a C-C beam, especially for the flexure mode. The quality factor of the operated mode decreased with increasing temperature, and changed drastically when the micromachined gyroscopes worked at higher temperatures. The probable cause was that the die attachment was softened as the temperature increased.
